# Programmable optoelectronic Ising machine for optimization of real-world problems

**DOI:** 10.1038/s41377-025-02100-9

**Published:** 2026-01-01

**Authors:** Zhewen Hu, Yanbo Ren, Yao Meng, Tiejun Wang, Yanchen Jiang, Miaomiao Wei, Ye Xiao, Zhentong Li, Ming Li

**Affiliations:** 1 State Key Laboratory of Optoelectronic Materials and Devices, Institute of Semiconductors, Chinese Academy of Sciences, Beijing, China; 2 Center of Materials Science and Optoelectronics Engineering, University of Chinese Academy of Sciences, Beijing, China; 3 School of Electronic, Electrical and Communication Engineering, University of Chinese Academy of Sciences, Beijing, China; 4 State Key Laboratory of Information Photonics and Optical Communications and School of Science, Beijing University of Posts and Telecommunications, Beijing, China; 5 Ising Intelligent Technology Co. Ltd., Beijing, China

**Keywords:** Microwave photonics, Fibre optics and optical communications

## Abstract

Ising machines offer a paradigm shift from traditional computing methods, tackling complex combinatorial optimization problems (COPs). Despite the proliferation of various Ising machine implementations, their application to solve real-world COPs has been limited. Here, we introduce a high-performance optoelectronic Ising machine (OEIM), based on optoelectronic parametric oscillators, that represents a significant advancement in this field. With 4096 Ising spins, arbitrary coupling capabilities, and unparalleled long-term stability, our OEIM outperforms traditional computing approaches in both accuracy and speed. By solving the benchmark maximum cut problem, we demonstrate its superior performance. More importantly, we apply the OEIM to a real-world traffic optimization problem, using real traffic data and a classical traffic model, and achieve results that far surpass those of conventional computers. This work not only validates the OEIM’s capability to solve complex practical challenges but also heralds a new era in real-time traffic management, where high-performance optoelectronic Ising machines enable rapid and efficient solutions to critical societal issues.

## Abstract (web summary)

High-performance optoelectronic Ising machine with 4,096 spins achieves superior accuracy, speed, and stability, solving benchmark and real-world traffic optimization problems beyond conventional computing.

## Graphical abstract

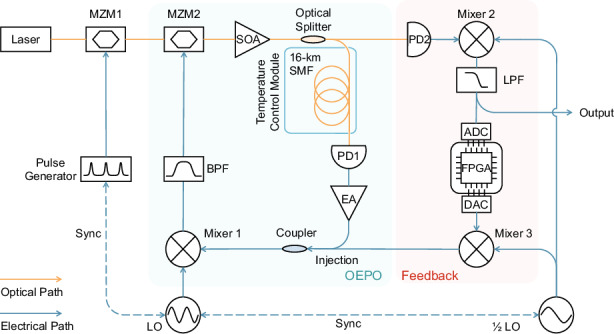

## Introduction

Combinatorial optimization problems (COPs), prevalent across domains including finance^1^, transportation^2^, synthetic biology^3^, and artificial intelligence^4^, pose formidable challenges due to their nondeterministic polynomial time (NP)-hard or NP-complete nature^5^. These problems pose challenges for efficient solutions on conventional Von Neumann computers, prompting the development of numerous heuristic algorithms^6^. A breakthrough approach is to map these COPs onto the minimization of Ising energy in the Ising model^7^. To realize such computations, various technological implementations of Ising machines have been proposed, including optical Ising machines^8–20^, Ising machines based on quantum adiabatic theorem^21–24^, and on-chip Ising machines realized through the design of specialized circuits^25–31^. Among them, coherent Ising machines (CIMs) utilizing degenerate optical parametric oscillators (DOPOs) have gained attention for their scalability and programmability^8–15^. Despite demonstrating advantages over conventional computers in solving mathematical problems like the maximum cut (MAX CUT)^32–34^, the potential of Ising machines in tackling real-world COPs remains largely unexplored.

Real-world COPs, characterized by extensive variables, intricate relationships, and stringent efficiency requirements, demand Ising machines with long-term stability, programmability, and scalability^35^. CIMs and superconducting quantum annealers, such as D-Wave’s systems, have ventured into solving real-world problems^15,36,37^. However, their high costs, demanding operating conditions, and limited continuous operation hinder widespread practical adoption.

**Fig. 1:**
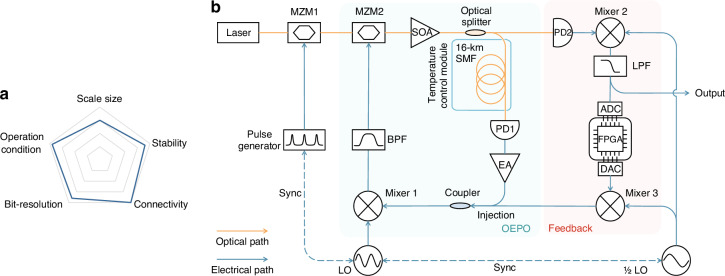
Performance radar chart and experimental setup of OEIM. **a** Performance radar chart illustrating the capabilities of OEIM, see Supplementary Table S3 for detailed comparison with other systems. **b** Schematic of the experimental setup. Ising spins are represented by the binary phase of OEPO pulses, and arbitrary connections between spins are established via an FPGA-based feedback loop. Key components include MZM (Mach-Zehnder modulator), SOA (semiconductor optical amplifier), SMF (single-mode fiber), PD (photodetector), EA (electrical amplifier), LO (local oscillator), BPF (bandpass filter), LPF (lowpass filter), ADC (analog-to-digital converter), FPGA (field-programmable gate array), and DAC (digital-to-analog converter)

Based on our original optoelectronic parametric oscillator (OEPO), our team previously proposed an Ising machine with the delay-line scheme^35,38^. This Ising machine implemented fixed-pattern spin-spin coupling in the optical domain via a wavelength-division multiplexed optical delay-line network, successfully solving specific MAX CUT problems. However, this coupling method has limitations in terms of spin connectivity and bit-resolution, making it insufficient to meet the requirements of practical applications. In this work, building upon our previous research and leveraging the advantages of optical waves and microwaves, we transferred the spin-spin coupling from the optical domain to the microwave domain. By employing a feedback loop with a field-programmable gate array (FPGA), we achieved arbitrary coupling between Ising spins with high bit-resolution, thus developing a large-scale, programmable, and stable optoelectronic Ising machine (OEIM) for practical applications. This OEIM, equipped with 4096 Ising spins, operates reliably at room temperature and exhibits stable long-term performance along with arbitrary coupling capabilities, as shown in Fig. 1a. By comparing its performance to simulated annealing (SA) in solving the MAX CUT problem, we underscore its superiority. Furthermore, we demonstrate the OEIM’s capacity to tackle a traffic optimization problem using real data, surpassing conventional computers in both solution quality and speed. This achievement highlights the OEIM’s practical potential in resolving complex real-world COPs.

## Results

### Principle of OEIM

Figure 1b shows a schematic of the OEIM; see Materials and methods for detailed information. In our scheme, OEPO pulses with phases locked at 0 or π serve as Ising spins. The mixer functions similarly to the nonlinear crystal in the CIM^11^, forming the parametric oscillation^38^ process and establishing binary-phase oscillation. Since microwaves have a lower frequency than optical waves, which makes microwave pulses easier to manipulate and more compatible with electronic devices, we implemented spin-spin coupling in the microwave domain using an FPGA-based feedback loop. The FPGA calculates the required feedback strength, while a frequency conversion circuit composed of mixers and filters generates the feedback signal. By sending the feedback signal back to the corresponding OEPO pulses, arbitrary programmable coupling between Ising spins is achieved (see Methods for details). The system energy of the OEIM constructed using this scheme takes the same form as the Ising Hamiltonian. The Ising Hamiltonian, in the absence of an external magnetic field, is expressed as^39^

1 $$H=-\mathop{\sum }\limits_{1\le i < j < N}{J}_{{ij}}{\sigma }_{i}{\sigma }_{j}$$

where *σ* denotes the Ising spins taking values of −1 or 1, *J* is the Ising coupling matrix, *N* is the number of spins, and *H* is the Ising energy. Based on the principle of minimum power dissipation, the Ising machine converges to the minimum energy state^35,40^, where the spin set corresponds to the lowest Ising energy defined by the *J* matrix (see Supplementary Section 1).

### Solving the MAX CUT problem

To assess the OEIM’s performance, we applied it to the MAX CUT problem, which is mathematically equivalent to the Ising model^41^. The cut value in the MAX CUT problem maps to the Ising energy in the Ising model, with the transformation formula given by

2 $${cut\; value}=-\frac{1}{2}(\mathop{\sum }\limits_{1\le i < j < N}{J}_{{ij}}+H)$$

For a given *J* matrix, the minimum Ising energy and the maximum cut value share the same spin set.

First, to compare the OEIM’s computation time with a standard Von Neumann architecture computer, we generated a fully connected graph, I4096, with 4096 nodes and 8,386,560 edges, where all edge weights are either 1 or −1 (see Supplementary Section 4). Since obtaining a theoretical exact solution for large-scale MAX CUT problems in polynomial time using a computer has been proven infeasible^42^, a target value is needed to determine the effectiveness of the solution. Therefore, we employed the SG3 (Sahni-Gonzales 3) algorithm, which is commonly used to find targets for large-scale MAX CUT problems^43^ (see Supplementary Section 5). To evaluate the computation time of the OEIM, we selected the simulated annealing (SA) algorithm for comparison, as SA is known for effectively solving various types of COPs^44^. Due to the parameter sensitivity of the SA algorithm, it can exhibit different advantages under different parameter settings. By performing parameter sweeps, we identified configurations that enable the algorithm to converge in a shorter time (referred to as the speed mode) and configurations that allow for higher cut values (referred to as the accuracy mode). To ensure a fair comparison between the OEIM and the SA algorithm, we applied different configurations of SA in different comparative experiments. Specifically, the SA algorithm was set to speed mode when comparing computation time, and to accuracy mode when comparing solution accuracy (see Materials and Methods and Supplementary Section 6).

**Fig. 2:**
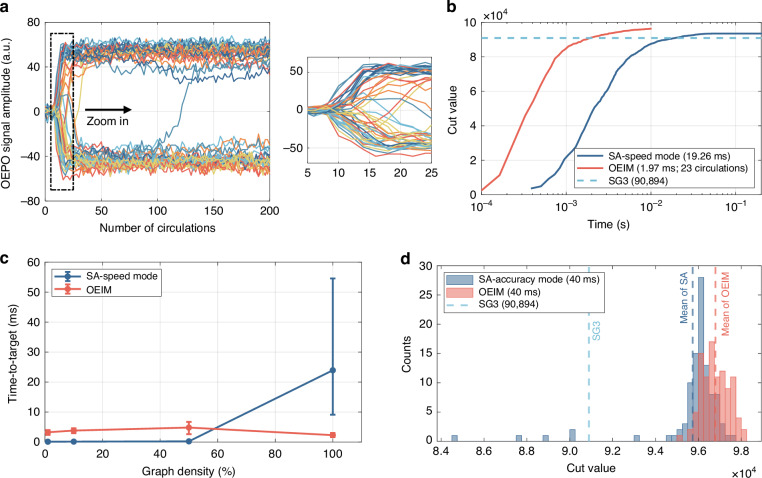
Solving the MAX CUT problem with OEIM and comparison with SA. **a** Evolution of spin amplitude with increasing number of circulations for the I4096 problem. Inset shows rapid bifurcation of Ising spins at the onset of evolution. **b** Temporal evolution of the cut value obtained by OEIM (red curve) and SA in speed mode (blue curve) for the I4096 problem. The dashed line indicates the target value (90,984) attained by SG3 (see Supplementary Section 5). **c** Time-to-target for 4096-node graphs under varying graph densities (1%, 10%, 50%, 100%) using OEIM and SA in speed mode. **d** Histogram of cut values obtained by OEIM and SA in accuracy mode for the I4096 problem. The best and mean cut values achieved by OEIM are 98,236 and 96,794, respectively, while those for SA are 97,564 and 95,731

We first used the OEIM to solve the I4096 problem and compared the solving process with the SA algorithm in speed mode. The *J* matrix corresponding to the I4096 graph is loaded into the OEIM system via an FPGA for solving, with amplitude signals of the Ising pulses collected every two cycles. Figure 2a illustrates the evolution of spin amplitudes with respect to the number of circulations while solving the I4096 problem by the OEIM. The OEIM rapidly bifurcated within the first 23 circulations, achieving a significant drop in Ising energy (i.e., a rapid increase in the cut value). Around 40 circulations, most spin amplitudes saturated, while a few pulses continued to flip, enabling the system to explore lower energy states. Figure 2b shows the temporal evolution of the cut value for solving the I4096 problem using the OEIM and the SA in speed mode. The OEIM reached the target set by SG3 after 23 circulations, taking 1.97 ms, while SA took 19.26 ms, approximately 10 times longer. The OEIM achieved a final cut value of 96,386, higher than SA’s stable cut value of 93,475, indicating the OEIM’s efficiency.

We investigated the time-to-target of the OEIM and SA under different density conditions. We used 4096-node graphs (see Supplementary Section 4) with graph densities of 1%, 10%, 50%, and 100% to solve the MAX CUT problem on both the OEIM and SA, recording the time taken to reach the target set by SG3. For each graph density, we ran the OEIM and SA in speed mode 100 times each. We performed a statistical analysis of the solution results and plotted an error bar plot, where the top and bottom of each bar represent the maximum and minimum computation times within each group, respectively. The mean computation times for different densities were connected to reveal the overall trend, as shown in Fig. 2c. SA had an advantage in computation time at lower graph densities, but its time-to-target increased significantly as graph density increased. Conversely, the OEIM maintained a relatively constant time-to-target across different graph densities, which means its computation time does not increase with higher graph densities. Additionally, at 100% graph density, the SA algorithm exhibited significant fluctuations in the time required to reach the target solution, whereas the OEIM showed much smaller variations. These two observations together demonstrate that the OEIM offers both faster and more stable performance when solving high-density MAX CUT problems. Moreover, we believe that in the OEIM system, as the problem size increases, the number of required spins grows accordingly, resulting in a linear increase in computation time. In contrast, on conventional computers, computation time typically increases faster than linearly with problem size^5^. Therefore, we anticipate that the speed advantage of OEIM will become increasingly prominent as problem complexity scales up.

We also compared the statistical distribution of multiple solution results for the I4096 problem between the OEIM and SA in accuracy mode (see Supplementary Section 6). The computation results of the OEIM and SA at 40 ms were utilized for statistical analysis. At this point, the OEIM had completed approximately 488 circulations, with the Ising spin amplitudes attaining saturation, and the SA had also nearly converged. Figure 2d illustrates the distribution of cut values obtained by the OEIM and SA 100 times each (for more details, see Supplementary Section 7). All OEIM results reached or exceeded the target set by SG3, while a small portion of SA results did not meet the target. The best and average cut values achieved by the OEIM were 98,236 and 96,794, respectively, both surpassing the best and average cut values of SA within the same timeframe. Furthermore, we extended the computation time of the SA algorithm in accuracy mode to 2 s and conducted 100 runs. The results were then compared with those obtained under the 40 ms setting described above, as shown in Table 1. With sufficient computation time, the SA algorithm achieved cut values higher than those of the OEIM. This indicates that within a limited computation time, the OEIM exhibits an advantage over SA in both computation time and accuracy; however, given sufficient computation time, SA still retains an edge in achieving superior solution results.

**Table. 1:** Best and mean cut values obtained from 100 runs each by the OEIM and SA (in accuracy mode)

	Best	Mean
OEIM (40 ms)	98,236	96,794
SA (40 ms)	97,564	95,731
SA (2 s)	99,129	97,943

### Evaluating stable oscillation time of OEIM

To evaluate the stability of the OEIM, we discontinued the injection of feedback signals, resulting in 4200 Ising spins being randomly distributed. During the stable oscillation of the OEIM, this random Ising spin sequence remained invariant; that is, the {+1, −1} random sequence corresponding to all Ising spins in each loop cycle did not alter with prolonged oscillation time. Conversely, when the system ceased to oscillate stably, the Ising spin sequence changed, differing from the initial sequence. The time elapsed from the establishment of the Ising spin sequence to the discernible change in the Ising spin sequence is denoted as the system’s stable oscillation time.

To monitor the stable oscillation time of the OEIM, we employed high-speed acquisition equipment to capture the Ising spin sequence every 15 s. Subsequently, we calculated the Pearson correlation coefficient *r* between the initial and current Ising spin sequences. When the coefficient *r* exceeds 0.95, the two sequences exhibit a high degree of correlation^45,46^, indicating that the state difference of the OEIM at these two moments is minimal and the system maintains stable oscillation.

**Fig. 3:**
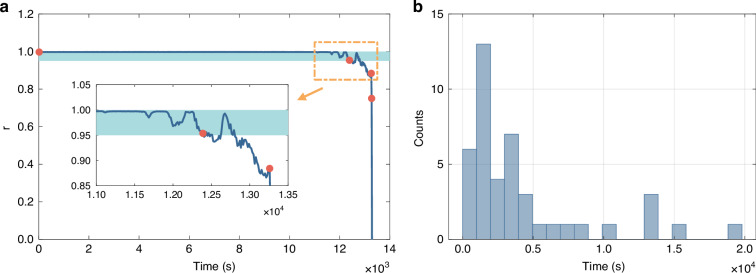
Stable oscillation time of OEIM. **a** Temporal variation in the Pearson correlation coefficient *r* of OEIM, with characteristic points marked in red. The inset depicts fluctuations in *r* curve as OEIM approaches the end of stable oscillation. **b** Histogram of stable oscillation time of the OEIM with temperature control module

Figure 3 presents a typical process of stable oscillation and the statistical results from multiple measurements. Figure 3a shows a typical temporal variation of the Pearson correlation coefficient *r* of OEIM, with the inset displaying the detailed fluctuation of the curve as the system approaches instability. To better illustrate the entire stable oscillation process, several characteristic points are marked with red dots in Fig. 3a. Starting from the initial state, the first characteristic point, the Pearson correlation coefficient *r* remains close to 1.0, indicating good stability of the OEIM. From the second characteristic point onward, the coefficient *r* begins to fluctuate but remains above 0.95, suggesting that the system still maintains a stable oscillation state. At the third characteristic point (12,390 s), the coefficient *r* is 0.955, but at the subsequent measurement point, it drops below 0.95, indicating that the system has lost its stable oscillation state. Therefore, we conclude that the stable oscillation time of the OEIM in this instance is 12,390 s (3.4 h). To further demonstrate the stability of the OEIM, we present the statistical histogram of stable times from multiple measurements in Fig. 3b. We randomly selected measurement start times and conducted a total of 43 measurements. The histogram of the stable oscillation times is shown in Fig. 3b. The average stable oscillation time of the OEIM was 4250 s (1.1 h), with the best stable oscillation time of 19,785 s (5.5 h).

### Solving a real-world traffic optimization problem

**Fig. 4:**
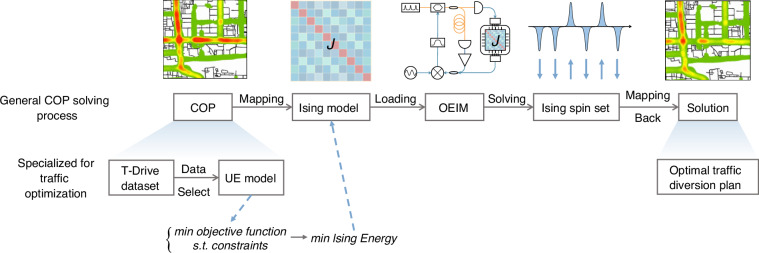
Solving process for general COP and traffic optimization problems. **1** Mapping of the COP to the Ising model. **2** Loading the *J* matrix from the Ising model into OEIM via FPGA. **3** Evolution of OEIM to obtain the Ising spin set. **4** Mapping the Ising spin set back to the COP to yield the optimized solution. For traffic optimization, data from the T-Drive dataset is used, the UE model is converted to an Ising model, and the optimized solution represents the optimal traffic diversion plan

After validating the performance of the OEIM through its application to MAX CUT problems, we further sought to apply the OEIM to solve COPs encountered in real-world scenarios (the solving process is depicted in Fig. 4). Specifically, we chose a particular traffic optimization problem as a representative example of a COP. By addressing this problem with the OEIM, we aimed to assess its potential for practical applications.

In this study, we selected the northbound section of the West Second Ring Road in Beijing, from Beijing West Railway Station to Gulou Avenue, along with the surrounding branch roads, as the study area. This region covers approximately 50 km^2^ and suffers from significant congestion during peak hours. We selected 1200 vehicles traveling on this road segment from the T-Drive dataset, a well-known open-source dataset based on real GPS data from vehicles^47–49^. The vehicles were divided into 160 groups, with each group having three alternative diversion routes. Additionally, to better reflect real-world conditions, we included 1500 baseline vehicles that did not participate in the diversion.

The User Equilibrium (UE) model is commonly employed in traffic optimization to strategically plan vehicle routes, thereby achieving traffic diversion and alleviating congestion^50,51^. Mathematically, the UE model can be transformed into a form resembling the Ising model, where the objective function to be optimized in the UE model is equivalently converted into the Ising energy in the Ising model. When the Ising energy reaches its minimum value, the objective function in the UE model is optimized, and the corresponding Ising spin set represents the optimal traffic diversion plan. Therefore, we adopted the UE model to describe the selected optimization problem.

We used three Ising spins to represent the route choice for each vehicle group: a spin-up indicates that the group selects the route, and vice versa. Furthermore, we had to satisfy the constraint that each group could only choose exactly one route from the three alternatives. An additional five Ising spins were used as auxiliary spins to transform the typical Ising model with an external magnetic field into the zero-field Ising model solvable by the OEIM. Therefore, a total of 160 × 3 + 5 = 485 Ising spins were required for this model’s computation (see Supplementary Section 8).

**Fig. 5:**
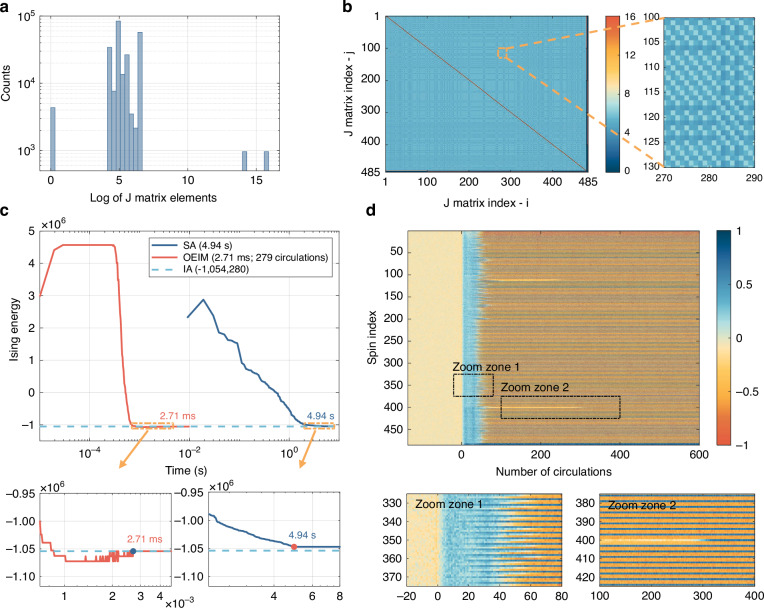
Solving the real-world traffic optimization problem with OEIM and comparison with SA. **a**, **b** Histogram and heatmap of the Ising coupling matrix elements for the traffic optimization problem. **c** Temporal evolution of Ising energy obtained using OEIM (red curve) and SA (blue curve). The dashed line denotes the reference Ising energy computed with IA (−1,054,280). Insets showcase the OEIM and SA curves nearing completion. **d** Amplitude evolution of each spin with increasing number of OEIM circulations. Zoom zone 1 displays the initial evolution, while zoom zone 2 captures the final stages

Unlike the Ising coupling matrix in the MAX CUT problem, where matrix elements only take values of 1, 0, or −1, the coupling matrix elements for real-world problems exhibit high bit resolution. This poses a significant challenge in configuring the feedback loop to effectively represent the high-precision information in the Ising coupling matrix (see Supplementary Section 9). Figure 5a, b show the distribution histogram and heatmap of the Ising coupling matrix elements (in log-scale) for the traffic optimization problem loaded into the FPGA. It can be observed that the amplitude of the matrix elements spans a wide range, with the maximum and minimum values differing by 16 orders of magnitude. To minimize information loss, we configured 16-bit resolution analog-to-digital converters (ADCs), digital-to-analog converters (DACs), and an FPGA in the feedback loop to obtain high-precision feedback signals (see Supplementary Section 2).

Similar to the approach used for solving the MAX CUT problem with the OEIM, we also needed a reference for the traffic optimization problem to compare the performance differences between the OEIM and computers. Although the traffic optimization problem is infeasible to compute the theoretical exact solution, which is the same as the large-scale MAX CUT problem, we can obtain a theoretically near-exact solution using the Incremental Assignment (IA) method (see Supplementary Section 10). The IA method is a classical non-equilibrium assignment method in the traffic field. Due to its use of Dijkstra’s algorithm for searching the shortest path in a weighted graph, it can yield high-quality solutions after a sufficiently long computation time and is widely used for traffic optimization^52^. We set the Ising energy value of −1,054,280, obtained via the IA method in 140 s, as the reference Ising energy. To compare the performance differences between the OEIM and computers in solving this practical problem, we developed a simulated annealing (SA) algorithm specifically optimized for this problem (see Supplementary Section 6).

We solved this problem using both the OEIM and the SA algorithm. Figure 5c shows the evolution of the Ising energy over time for the best solution obtained by the OEIM (in red) and SA (in blue), while Fig. 5d illustrates the change in the amplitude of each Ising spin with the number of circulations of the OEIM. As shown in Fig. 5c and zoom zone 1 of Fig. 5d, during the first 30 circulations, most of the spins in the OEIM evolved from noise to the spin-up state (with relatively small amplitudes), causing an increase in Ising energy. Between circulations 30 and 70, most of the Ising spins rapidly bifurcated and their amplitudes saturated, resulting in a sharp decrease in Ising energy until it slightly dipped below the reference Ising energy, as indicated in the first inset of Fig. 5c. This dip occurred because the objective constraint was not fully enforced yet. In the subsequent circulations, the few remaining Ising spins that had not yet completed their evolution were influenced by the objective constraint, eventually achieving a spin set corresponding to the reference Ising energy. As shown in zoom zone 2 of Fig. 5d, the last spin that had not completed its evolution flipped from negative to positive at circulation 279, causing the system’s Ising energy to converge to the reference Ising energy (with the amplitude of this spin saturated at around circulation 310). The OEIM took 2.71 ms (279 circulations) to evolve to the reference Ising energy of −1,054,280. In contrast, the SA algorithm took 4.94 s to converge to an Ising energy of −1,047,440, failing to reach the reference Ising energy shown in the second inset of Fig. 5c.

**Fig. 6:**
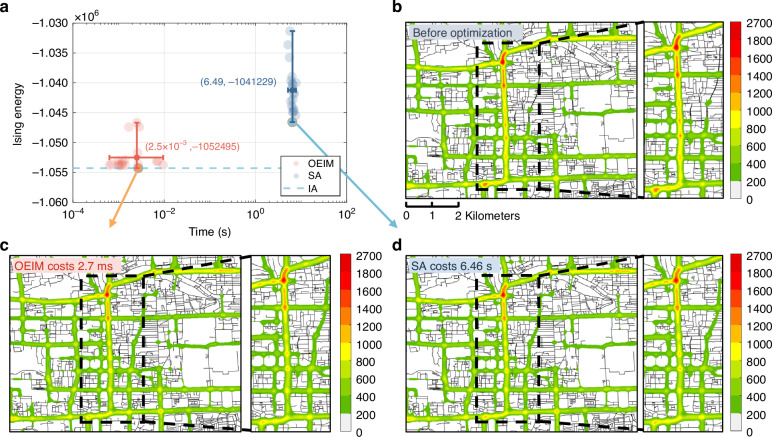
Multiple solutions for real-world traffic optimization and traffic heatmaps. **a** Comparison of computation time and final achieved Ising energy for multiple solutions obtained by OEIM (red dots) and SA (blue dots). **b** Traffic heatmap before optimization. **c**, **d** Traffic heatmaps after optimization by OEIM (2.7 ms) and SA (6.46 s), respectively

Both the OEIM and the SA algorithm were given sufficient time to run 20 times each. We compared their performance by statistically analyzing the time taken for the Ising energy to converge to a stable value (referred to as computation time) and the final achieved Ising energy as shown in Fig. 6a. We also used PySA, a publicly available SA algorithm developed by NASA^53^, to solve this problem and compared it with the OEIM (See Supplementary Section 11). In terms of accuracy, the OEIM achieved an average Ising energy of −1,052,495, which is lower than the average Ising energy of −1,041,229 achieved by our SA, and it is also lower than PySA. This indicates that the OEIM can obtain results closer to the optimal solution. Regarding computation time, the OEIM has an average computation time of 2.5 ms, which is three orders of magnitude faster than our SA algorithm with an average computation time of 6.49 s, and it is also faster than PySA.

We selected the best optimization results obtained from both the OEIM and SA and compared them with the pre-optimization state. The Ising spin sequences from the optimized results were mapped back to the route choices for each vehicle group. The traffic heat map visualizes road congestion by displaying the number of vehicles per unit density. In these heat maps, colors indicate the vehicle density: green represents fewer vehicles per unit density, while red indicates more vehicles per unit density. Figure 6b shows the traffic heat map before optimization. Figure 6c presents the traffic heat map after optimization by the OEIM, where it is evident that the extent of the red areas is significantly reduced, and many yellow areas have been downgraded to green, indicating a noticeable relief of traffic congestion. Figure 6d displays the traffic heat map after optimization by SA. While the overall optimization effect is similar to that of the OEIM, the OEIM only costs 2.7 ms, far shorter than the 6.46 s required by SA.

## Discussion

Similar to other oscillator-based simulated Ising machines, the OEIM (OEPO-based) presented in this work operates on the principle of minimum power dissipation, which makes it easier to escape local optima and find the global optimum^35,54,55^ Specifically, the Ising spins in the OEIM rapidly evolve from a random noise state to a state that satisfies the oscillator’s minimum power dissipation under the constraints of the problem loaded into the FPGA. The resulting set of Ising spins corresponds to the optimal solution of the problem.

In this work, we conducted various performance tests on the OEIM. In solving the MAX CUT problem, the OEIM demonstrated advantages over SA in terms of computation time (to achieve comparable solution quality), solution accuracy (under the same computation time), and robustness to problem complexity (i.e., less performance degradation with increasing complexity). Additionally, it exhibited a prolonged period of stable oscillation. For a specific real-world traffic optimization problem, the OEIM achieved a computation speed that was three orders of magnitude faster than SA, and the average results across multiple runs were also significantly better, delivering effective optimization for the given real-world problem. This indicates that the OEIM holds advantages over the SA algorithm running on conventional computers in solving both typical mathematical problems and complex real-world problems. Moreover, the OEIM enables spin-scale expansion through energy-efficient approaches such as extending the optical loop length and increasing the pulse repetition rate. Therefore, as the number of spins increases, the energy-efficiency advantage of OEIM over conventional computers is expected to become increasingly pronounced.

We qualitatively plotted the performance radar chart of OEIM in Fig. 1a. The radar chart clearly demonstrates that the OEIM proposed in this paper has comprehensive performance advantages, which is why it is capable of effectively solving real-world COPs. In recent years, various types of Ising machines have emerged, each with distinct features—for example, optical approaches such as CIM^14^ and SPIM^19^, quantum annealing systems like the D-Wave 2000Q^23^, and electronic chip-based approaches such as ROSC^30^ and SBM^56^. A detailed comparison between the OEIM and these systems is provided in Supplementary Section 12.

Despite the aforementioned advantages, the OEIM still has several areas for improvement. In the current setup, the OEIM computation process does not yet employ a refined pump gain control scheme. Theoretical analysis indicates that stronger pump gain leads to faster system convergence but limits exploration of the solution space. Conversely, operating near the threshold results in slower convergence, allowing more extensive exploration of the solution space and increasing the probability of finding better solutions^14,35^. Therefore, in future work, we plan to optimize the pump gain level and design a dynamic tuning strategy for OEIM to better balance computation time and solution quality. In addition, unlike the CIM, which uses DOPO pulses as Ising spins, the OEIM uses OEPO pulses with a longer wavelength as Ising spins. While this scheme inherently offers better stability, it also requires a longer loop length to generate spins of the same scale, leading to a longer circulation time. Looking ahead, we aim to realize larger-scale spin systems in OEIM by jointly increasing the pulse repetition rate and the optical loop length, while keeping the circulation time within practical limits. Furthermore, the OEIM is currently constrained by the limited memory capacity and data transfer bandwidth of the FPGA, which hinders the implementation of coupling feedback for a larger number of spins and results in the problem matrix transmission time of approximately 60 s. These limitations are anticipated to be mitigated through enhancements to the current programmable pulse-coupled architecture of OEIM.

In summary, this study demonstrates that OEIM can efficiently solve real-world COPs by successfully addressing a practical traffic optimization problem, showing clear advantages over conventional computers. This result lays a foundation for potential applications in logistics allocation, path planning, and drug discovery. Furthermore, the high-precision OEIM developed in this work is expected to overcome the compatibility limitations that previously existed between Ising machines and complex neural network architectures, thereby advancing its application in domains such as AI training acceleration. Collectively, these findings highlight both the practical value and future application potential of the proposed OEIM.

## Materials and methods

### Experimental setup

In our proposed scheme (Fig. 1b), the optoelectronic Ising machine (OEIM) is constructed from optoelectronic parametric oscillator (OEPO) and a programmable pulse coupling system (earlier referred to as the feedback loop). The optoelectronic oscillation cavity enters a degenerate state by injecting a local oscillation (LO) signal into it via a mixer, thereby generating OEPO pulses. As the gain is gradually increased from zero, the OEPO pulses rapidly emerge from noise and attain a steady state. In this steady state, the OEPO pulse phases lock randomly at either 0 or π relative to the LO signal phase, representing Ising spins in the up (0) or down (π) states, respectively. To generate a substantial number of Ising spins, we constructed large-scale OEPO pulses using 16 km of single-mode fiber, Mach-Zehnder modulators, electrical amplifiers, bandpass filters, and high-speed photodetectors. This setup forms an optoelectronic oscillation cavity with a loop delay of 84 µs. To mitigate the impact of temperature fluctuations, we implemented temperature feedback control on the long fiber (see Supplementary Section 3). A 50 MHz repetition rate pulse signal is modulated onto a 1550 nm pump laser, and a 20 GHz LO signal is injected into the cavity through a mixer, resulting in the generation of 4200 OEPO pulses. The programmable pulse coupling system based on a field-programmable gate array (FPGA) subsequently establishes arbitrary connections between these OEPO pulses, conferring flexibility and programmability to the OEIM.

### Details of the feedback loop

To facilitate interactions between these pulses, we developed an FPGA-based programmable pulse coupling system. This system detects Ising spin signals, computes feedback signals, and reintroduces these feedback signals into the OEPO cavity. Specifically, we utilize an optical splitter to extract a portion of the optical pulse signal energy from the OEPO cavity and convert it into electrical signals via photodetectors. These electrical signals are then demodulated using a mixer to obtain the binary phase information of the pulses, which corresponds to the Ising spins. Subsequently, the Ising spin pulses are sampled and quantized by an analog-to-digital converter (ADC). The system designates N of these pulses as computational spins (involved in the computation) and the remaining 4200−N pulses as idle spins for sequence synchronization. The FPGA stores the amplitudes of the N computational spins in an N × 1 vector, multiplies this vector by the preloaded Ising coupling matrix of the problem to generate feedback signals, and outputs these signals via a digital-to-analog converter (DAC). The feedback signals are then modulated onto a 10 GHz carrier and reinserted into the OEPO cavity through a microwave coupler, thereby coupling the pulses. It is crucial to ensure that the feedback signals align and couple with the established OEPO pulses within the cavity for effective subsequent oscillations. Therefore, meticulous attention is paid to pulse timing synchronization, delay matching, coupling strength, and other related factors to achieve precise control.

### Solving process for COPs

The OEIM’s process for solving combinatorial optimization problems (COPs) is outlined as follows (Fig. 4): First, the target COP is transformed into an Ising model. Subsequently, the corresponding Ising coupling matrix is loaded into the programmable pulse coupling system via the FPGA. The OEIM is then activated, enabling it to swiftly converge to a steady state (i.e., the optimal solution state for the problem) under the constraints imposed by the programmed pulse coupling system. Finally, the Ising spin sets within the OEIM are mapped back to the variables of the original COP. When tackling COPs of varying scales, different numbers of Ising spins within the same OEIM contribute to the computation, with the circulation time for each computation being directly proportional to the number of involved Ising spins.

### Simulated Annealing (SA) algorithms

To comprehensively compare the computational performance of the OEIM and the SA algorithms, this study employs problem-specific SA algorithms with carefully optimized parameters. For MAX CUT problems, we used an SA algorithm developed by the CIM’s team specifically for this problem class, implemented in C^12^. In addressing the traffic optimization problem, we independently developed a dedicated SA algorithm to achieve high-quality solutions and further applied PySA^53^, a widely adopted and advanced open-source SA implementation, for benchmarking purposes. The detailed experimental parameter settings for all SA algorithms are provided in Supplementary Section 6.

## Supplementary information

Supplementary Information

## Data Availability

The data that support the findings of this study are available from the corresponding author upon request.
