# Supplementary material for: Programmable optoelectronic Ising machine for optimization of real-world problems
Source: Light Sci Appl. 2026 Jan 1;15:6. doi: 10.1038/s41377-025-02100-9 (PMC12756251; doi:10.1038/s41377-025-02100-9)
Supplement: Supplementary file 1 — Supplementary Information [file 41377_2025_2100_MOESM1_ESM.pdf]

Supplementary Information for

Programmable optoelectronic Ising machine for optimization of real-world problems

Zhewen Hu[1,2,3,5], Yanbo Ren[1,2,3,5], Yao Meng[1], Tiejun Wang[4], Yanchen Jiang[4], Miaomiao Wei[1], Ye Xiao[1], Zhentong Li[1,2,3], Ming Li[1,2,3,*]

[1]State Key Laboratory of Optoelectronic Materials and Devices, Institute of Semiconductors, Chinese Academy of Sciences; Beijing, 100083, China.
[2]Center of Materials Science and Optoelectronics Engineering, University of Chinese Academy of Sciences; Beijing, 100190, China.
[3]School of Electronic, Electrical and Communication Engineering, University of Chinese Academy of Sciences; Beijing, 100049, China.
[4]State Key Laboratory of Information Photonics and Optical Communications and School of Science, Beijing University of Posts and Telecommunications; Beijing, 100876, China.
[5]These authors contributed equally to this work: Zhewen Hu, Yanbo Ren.
[*]Corresponding author. Email: ml@semi.ac.cn

Contents

Section 1 System Dynamics 3

Section 2 FPGA Configuration 3

Section 3 Solution for System Temperature Stability 4

Section 4 The Definition and Generation of the MAX CUT problem 6

Section 5 SG3 Algorithm 8

Section 6 SA Algorithm 9

Section 7 Multiple Runs of OEIM Solving the I4096 Problem 11

Section 8 Traffic Optimization: Detailed Approach for Modeling 11

Section 9 Traffic Optimization: Selection of Bit Resolution for the *J* Matrix 14

Section 10 Traffic Optimization: IA Algorithm 14

Section 11 Traffic Optimization: Comparison of OEIM and SA Algorithms 15

Section 12 Comparison with Other Ising Machine Schemes 16

References 19

Section 1 System Dynamics

During the computation process of the OEIM, the amplitude of the OEPO pulses increases from zero until it reaches saturation. The computation of the OEIM is completed when the amplitude of the OEPO pulses saturates. Therefore, the evolution of the OEPO pulses is the core principle of the OEIM's computation. We have established the dynamical equation for the evolution of OEPO pulses in the OEIM[1] as

$$\frac{ds_i}{dt} = G_0 J_1\left(\frac{\pi s_i}{V_\pi}\right)\frac{1}{s_i}\left[(\gamma+\beta)s_i + \sum\nolimits_{i<j\le N} kJ_{i,j}s_j\right] - s_i \qquad (S1)$$

where s_i represents the amplitude of the i-th OEPO pulse, G_0 is the overall gain of the system. $J_1(\cdot)$ denotes the first-order Bessel function of the first kind, β and γ is the frequency conversion coefficient and the leakage coefficient of the mixer in OEPO respectively. J is the Ising coupling matrix, and $kJ_{i,j}$ is the coupling coefficient between different OEPO pulses, where k is a constant representing the coupling strength. Under the condition of feedback loop coupling injection, the optoelectronic cavity oscillates, with OEPO pulses evolving from noise to a steady state. Through this process, the minimum energy state of the input Ising model is determined.

Section 2 FPGA Configuration

To achieve arbitrary coupling between Ising spins in the OEIM, we need to construct a programmable pulse coupling system capable of real-time acquisition and computation, as previously mentioned in the feedback loop. An FPGA and its accompanying ADC and DAC modules possess characteristics such as real-time acquisition, high-speed parallel computation, and stable computation time, which perfectly meet our requirements. Therefore, we utilize FPGA with ADC and DAC modules to build the programmable pulse coupling system. We store the coupling between Ising spins in the form of an Ising coupling matrix in the FPGA (XCKU115-FLVA1517 from Xilinx) to facilitate real-time computation of feedback signals. The high-speed ADC (ADS54J60 from TI) with an input bit resolution of 16 bits is used to convert the Ising spins from

analog signals to digital signals, which were then input into the FPGA for feedback signals computation. The high-speed DAC (DAC39J84 from TI) with an output bit resolution of 16 bits is used to convert the feedback signals computed by the FPGA back into analog signals.

Given the large scale and high precision of the Ising coupling matrix we use, significant storage space is required. Thus, a reasonable storage scheme for the FPGA must be planned to effectively store the matrix in the FPGA. The specific plan is as follows:

The Ising coupling matrix corresponding to the maximum cut (MAX CUT) problem mentioned in this work has each matrix element as 2 bits, occupying a storage space of $4096 \times 4096 \times 2\ \text{bit} = 32\ \text{Mbit}$. To accommodate the structure and capacity limitations of the FPGA storage units, the matrix elements are divided horizontally and vertically into 44 submatrices. Sixteen of these submatrices are stored in 16 (2×8) distributed RAMs (512×512) and computed using the first subroutine. The remaining submatrices are stored in 28 (7×4) block RAMs (1024×1024) and computed using the second to fourth subroutines.

The Ising coupling matrix corresponding to the traffic optimization problem has each matrix element as 24 bits, occupying a storage space of $485 \times 485 \times 24\ \text{bit} \approx 5.38\ \text{Mbit}$. To accommodate the structure and capacity limitations of the FPGA storage units, the matrix elements are divided vertically into 11 submatrices. All submatrices are stored in 11 block RAMs, each submatrix being $43 \times 512 \times 24\ \text{bit} \approx 0.50\ \text{Mbit}$. We use 5 subroutines to process the first 10 submatrices, with each subroutine handling 2 submatrices, and the last submatrix is processed by a single subroutine. Finally, the results from all subroutines are output sequentially as the computation result of the Ising coupling matrix.

Section 3 Solution for System Temperature Stability

The OEIM utilizes high-Q values provided by long optical fibers within an optoelectronic hybrid cavity and employs dense time division multiplexing technology to achieve up to 4200 Ising spins. These Ising spins need to maintain a certain level of phase coherence. The longer the system operates stably, the more Ising spins are accumulated within the cavity for cyclic

computation, leading to an increased number of stable iterative cycles. This indicates that the OEIM will have an extended period of stable operation.

For the OEIM, phase perturbations are primarily determined by the microwave carrier frequency ω and the jitter in the cavity delay (Δt):

$$\Delta\phi = \omega\Delta t \qquad (S2)$$

The delay jitter is mainly determined by the effective optical path length L of the optical fiber

$$\Delta t = \frac{L}{c} \qquad (S3)$$

where c is the speed of light.

The effective optical path length is determined by the physical length l and the refractive index n , both of which are influenced by temperature. However, compared to the changes in physical length due to thermal expansion and contraction, the variation in n is more significant, on the order of 10^{-7} °C^{-1}. In summary, the longer the optical fiber, the greater the effective optical path length, and the more pronounced the phase jitter induced by temperature variations.

For an optical frequency of 100 THz, to keep phase perturbations within the 0.2π range, the effective optical path length variation must be confined to 300 nm. Such a 100-nm scale change in the optical path length can be caused not only by temperature fluctuations but also by uncontrollable environmental vibrations.

However, since the OEIM uses a microwave carrier, the phase change caused by variations in the effective optical path length is not as significant. When the effective optical path length changes by 3 mm, it introduces a 10 ps delay jitter within the cavity, leading to 0.2π phase changes. This tolerance is four orders of magnitude lower than that of a purely optical link. Due to the microwave loading of the Ising spins in the OEIM, it inherently possesses a long coherence time. Therefore, we only need to consider the phase perturbations caused by environmental temperature changes.

We tested the temperature variations in the experimental environment, finding that the day-night temperature difference can reach 7 °C. This temperature fluctuation can cause a change in the effective optical path length of about 10 cm for the 16 km long optical fiber within the Ising machine's cavity. To address this, our team designed a fiber temperature control module, which

can maintain the cavity temperature within a specified range of ± 0.5 ℃. By placing the long optical fiber within this temperature control module, we can effectively mitigate the impact of environmental temperature changes on the system. After implementing the temperature control module, the stable oscillation time of the OEIM increased significantly.

Section 4 The Definition and Generation of the MAX CUT problem

The MAX CUT problem is a fundamental problem in graph theory and combinatorial optimization. Given an undirected graph $G = (V, E)$ where V is the set of vertices and E is the set of edges, the objective of the MAX CUT problem is to partition the vertex set V into two disjoint subsets V_1 and V_2 such that the number of edges between V_1 and V_2 is maximized. In other words, the goal is to find a cut (V_1, V_2) that maximizes the number of edges crossing the partition.

Based on the definition of the Max Cut problem described above, we generated graph structures for Max Cut problems with a specified number of vertices and graph density, where edge weights are chosen from $\{-1, 0, 1\}$ using a random algorithm. The pseudocode for generating the Max Cut problem is provided below.

Algorithm 1 *Generation of MAX CUT Problems*

Input: number of vertices n, graph density parameter p.
Output: the Ising coupling matrix for a MAX CUT problem J.
$values = [-1, 0, 1]$
Generate empty matrix J
for $i = 0$ to $n - 1$ **do**
for $j = 0$ to $n - 1$ **do**
$J[i][j] = random(values)$ // create a random matrix
end for
end for
for $i = 0$ to $n - 1$ **do**
for $j = 0$ to $n - 1$ **do**
$r = random\ in\ range\ (-1, 1)$
if $r > p$ **then**
$J[i][j] = 0$ // create a matrix with assigned graph density

else if $r < -p$ **then**
$J[i][j] = -1$
else
$J[i][j] = 1$
end else if
end if
end for
end for
$J = J + J' - \text{diagonal}(J)$ // symmetrize and remove diagonal
return J

Specifically, to test the computation time and solution accuracy of the OEIM, we generated a fully connected graph with 4,096 nodes. This graph, named I4096, is an undirected graph with 8,386,560 edges, where all edges have weights of either 1 or -1. By adjusting the density parameter p, we also generated MAX CUT problems with 1%, 10%, 50%, and 100% graph densities to test the performance of the OEIM. Fig. S1 presents the heatmaps of the elements of these matrices.

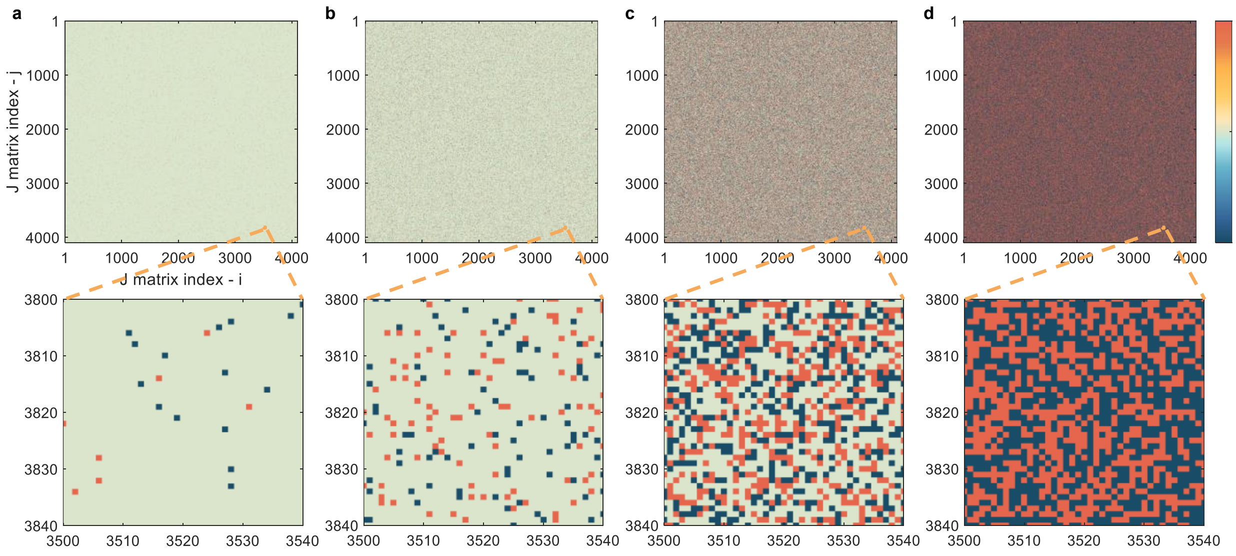

Fig. S1 | Heatmaps of Ising coupling matrix elements for 4096-node graphs with different graph densities. a-d, Heatmaps of the Ising coupling matrix with graph densities of 1%, 10%, 50%, and 100%, respectively. Insets below each heatmap depict local structural details.

Section 5 SG3 Algorithm

Given that obtaining an exact theoretical solution for large-scale MAX CUT problems in polynomial time using a computer is infeasible[2], it is crucial to establish a target for validating solutions. In prior research on CIM, the Goemans-Williamson semidefinite programming (GW-SDP) algorithm, which guarantees solutions with an expected value of at least 87.8% of the optimal value for MAX CUT problems[3], and the Sahni-Gonzales (SG) algorithm, a greedy heuristic[4], have been employed as targets[5-7]. Moreover, studies have shown that the SG algorithm can produce results close to those of GW-SDP in a shorter amount of time when solving large-scale MAX CUT problems. Considering both computational efficiency and solution accuracy, we opted to use an enhanced version of the SG algorithm, known as SG3, to establish the target[8]. The pseudocode for SG3 algorithm is as follows:

***Algorithm 2** SG3*

Input: A weighted graph $G = (V, E, w)$.
Output: Maximum value cut_value.
Choose v_1 and v_2 randomly.
Put v_1 in V_1 and v_2 in V_2.
$V' \leftarrow V \backslash \{v_1, v_2\}$
for $i = 0$ to $n - 2$ **do**
for j in V' **do**
Calculate $w(j, V_1)$ when v_j in V_2.
Calculate $w(j, V_2)$ when v_j in V_1.
$score(j) = |w(j, V_2) - w(j, V_1)|$
end for
Find the j' that maximizes $score(j)$.
$V' \leftarrow V' \backslash v_j$
if $w(j', V_2) > w(j', V_1)$ **then**
Put $v_{j'}$ in V_1
else
Put $v_{j'}$ in V_2
end if
$cut\_value = cut\_value + max\{w(j', V_2), w(j', V_1)\}$
end for
return cut_value

Section 6 SA Algorithm

In this work, we compared the performance of three SA algorithms with the OEIM. When solving MAX CUT problems, we used SA (CIM) in both Accuracy Mode and Speed Mode for comparison with the OEIM, as shown in Fig. 2B, 2C, and 2D. For the traffic optimization problem, we compared MATLAB SA with the OEIM, resulting in Fig. 5C and Fig. 6A. Additionally, we compared MATLAB SA and PySA with the OEIM, which is presented in Fig. S3.

6.1 SA(CIM)

We utilized the efficient simulated annealing (SA) algorithm referenced from the work on CIM[6] to solve the MAX CUT problem discussed in this paper and compared its performance with the OEIM. In our work, we used a standard SA algorithm implemented in C for efficiency, running on a CPU (AMD Ryzen 5 5600H, 3.30 GHz). To enable the SA algorithm to operate in either a higher accuracy mode or a faster convergence speed mode, we optimized the time scaling factors (T) and temperature scaling factors (β_0). T is the exploration–exploitation trade-off parameter that determines the convergence speed of the SA algorithm, while β_0 is the sensitivity parameter in the Metropolis criterion of SA, which adapts to the energy landscape of the problem. Together, these two parameters jointly influence the solution speed and accuracy of the SA algorithm. Specifically, we swept T from 1000 to 8000 in steps of 1000 and β_0 from 1.0 to 6.0 in steps of 1.0 to find the optimal parameter combinations for SA. The optimal parameters for different SA modes are shown in Table S1. Figure 2B, 2C illustrate the SA algorithm operating in speed mode, while Fig. 2D shows it operating in accuracy mode.

Mode	Time Scaling Factor (T)	Temperature Scaling Factor (β_0)
Accuracy Mode	6000	4.0
Speed Mode	200	2.0

Table S1 | T and β_0 for the different modes of SA.

6.2 MATLAB SA

To efficiently solve the traffic optimization problem, we developed a targeted SA algorithm based on MATLAB. The pseudocode for this SA algorithm is as follows:

***Algorithm 3** SA for Traffic Optimization Problems*

Input: Ising matrix J of traffic assignment.
Output: the final Ising spin set σ, the final Ising energy E.
Initialize Ising spin variable σ_0 and temperature T_0.
Calculate initial energy E_0.
loop
randomly flip a spin from σ_0, get σ'
calculate new energy E' and $\Delta E = E' - E_0$
if $\Delta E \leq 0$
$\sigma = \sigma'$ and $E = E'$
else $\exp(-T \times \Delta E) < \mathrm{rand}(1)$
$\sigma = \sigma'$ and $E = E'$
end if
$T = T_0 \times \log_2(1 + i/n)$ // n is the number of iterations, i represents the i-th
end loop
return σ E

6.3 PySA

We also used PySA, a publicly available SA algorithm developed by NASA, to solve the traffic optimization problem[9]. To ensure that PySA operated under reasonable parameter settings, we optimized two key parameters: the number of iterations (n) and the temperature interval (ΔT). Specifically, we conducted a parameter sweep for n within the range [500, 1000, 1500, 2000, 2500, 3000] and for ΔT within the range [0.00001, 0.0001, 0.001, 0.01, 0.1, 1] to determine the optimal parameter combination. To balance computation time and solution quality, we ultimately selected $n = 2000$ and $\Delta T = 0.1$ as the operating parameters for the PySA algorithm.

Section 7 Multiple Runs of OEIM Solving the I4096 Problem

To further evaluate the solution quality of the OEIM, we used it to solve the I4096 problem 300 times and conducted a statistical analysis of the results. Fig. S2 presents the histogram of the OEIM computation results. All OEIM results exceeded the target set by SG3, with an average cut value of 95,670.

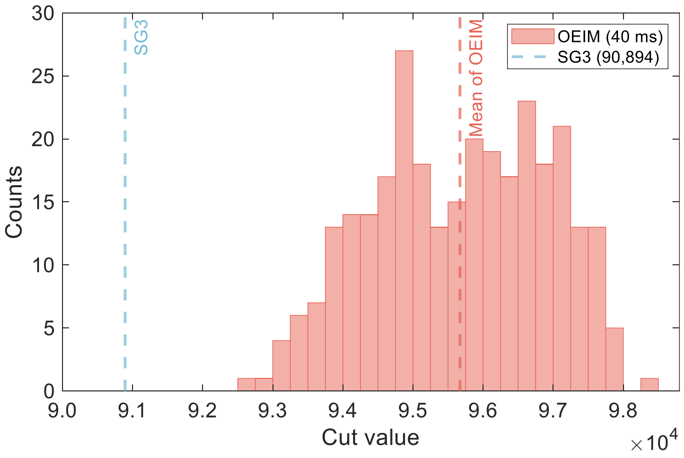

Fig. S2 | Histogram of 300 runs of OEIM solving the I4096 problem.

Section 8 Traffic Optimization: Detailed Approach for Modeling

Traffic assignment is the process of distributing traffic demand between origin-destination (OD) pairs across an existing road network based on travelers' route choice criteria, which aims to minimize overall travel impedance. This process often involves solving a combinatorial optimization problem (COP). The foundation of traffic assignment lies in Wardrop's equilibrium principle, which defines two key concepts: user equilibrium (UE) and system optimal (SO)[10]. In real-world scenarios, UE is more commonly considered, as it reflects individual drivers' tendency to choose routes that minimize their own travel time.

The function of the UE model is given by

$$\min: Z(X) = \sum_{s\in S} \int_0^{x_s} t_s(w)dw \qquad s.t. \begin{cases} \sum_r f_r^{od} = f_{\text{all}} \\ f_r^{0d} \geq 0 \end{cases} \tag{S4}$$

Here, s denotes a road segment, and S represents the set of all road segments. The variable x_s indicates the traffic flow on segment s, while t_s is the impedance function associated with segment s. Additionally, w is the integral variable of the traffic flow on segment s. f_r^{od} is the traffic flow on the route r from origin o to the destination d, f_{all} is the total traffic demand between origin o to the destination d. The most commonly used impedance function is the Bureau of Public Roads (BPR) function: $t_s = t_0^s\left[1+\alpha\left(\frac{x_s}{x_{\text{max}}^s}\right)^\beta\right]$. t_0^s represents the free-travel time of section s. x_{max}^s represents the maximum traffic limit for section s. The impedance coefficients α and β are specified as 0.15 and 4, respectively. By substituting BPR function into Eq. (S4), we can get

$$\begin{aligned}\min: Z(X) &= \sum_{s\in S}\int_0^{x_s} t_s(w)dw \\ &= \sum_{s\in S}\int_0^{x_s} t_0^s[1+\alpha\left(\frac{w}{x_{\text{max}}^s}\right)^\beta]dw \\ &= \sum_{s\in S}\int_0^{x_s} d\left\{t_0^s\left[w_s+\frac{\alpha}{\beta+1}\left(\frac{w}{x_{\text{max}}^s}\right)^{\beta+1}\right]\right\} \\ &= \sum_{s\in S} t_0^s\left[x_s+\frac{\alpha}{\beta+1}\left(\frac{x_s}{x_{\text{max}}^s}\right)^{\beta+1}\right] \end{aligned} \quad \text{(S5)}$$

Our study takes $\alpha = 0.15$, $\beta = 0.15$, so Eq. (S5) is a fifth-order function. We use the method of quadratic function approximation for order reduction.

$$\begin{aligned}\min: Z(X) &= \sum_{s\in S} t_0^s\left[x_s+\frac{\alpha}{\beta+1}\left(\frac{x_s}{x_{\text{max}}^s}\right)^{\beta+1}\right] \\ &\approx \sum_{s\in S} t_0^s(a_s{x_s}^2+b_sx_s+c_s) \end{aligned} \quad \text{(S6)}$$

Here, a_s, b_s, and c_s are the parameters of the quadratic approximation function. The objective function of traffic assignment is represented as a quadratic function for finding the minimum value, which can be solved by Ising machine.

The details of modeling the traffic assignment problem as a COP are as follows. Traffic demand is divided into N groups, each consisting of k vehicles, and M alternative routes are provided for each group. Each group can only choose one route from the alternative routes. Binary decision variable $q_{ij} \in \{0,1\}$ represents whether the i-th group selects the j-th route ($q_{ij} = 1$ it

selected, $q_{ij} = 0$ otherwise). So, the traffic flow on segment s is $x_s = \left(k \sum_{i,j} q_{ij}\, \delta_{s,ij} + x_0\right)$, where $\delta_{s,ij}$ is the indicator function of segment and route, x_0 is the initial traffic flow. The objective function for the traffic assignment from the perspective of combinatorial optimization is formulated as

$$\min: f\left(q_{ij}\right) = \sum_{s \in S} t_0^s (a_s {x_s}^2 + b_s x_s + c_s) + \lambda \sum_i^N (\sum_j^M q_{ij} - 1)^2 \qquad (S7)$$

where, λ represents the constraint coefficient. Using the spin $\sigma_{ij} \in \{-1,1\}$ replace q_{ij}, so $x_s = [k \sum_{i,j} (\frac{\sigma_{ij}+1}{2})\, \delta_{s,ij} + x_0]$. The objective function is

$$\min: f\left(\sigma_{ij}\right) = \sum_{s \in S} t_0^s (a_s {x_s}^2 + b_s x_s + c_s) + \lambda \sum_i^N [\sum_j^M (\frac{\sigma_{ij}+1}{2}) - 1]^2 \qquad (S8)$$

Expanding Eq. (S8) reveals that it consists of quadratic terms, linear terms, and constant terms. By disregarding the constant term, the linear term can be converted into a quadratic term through the introduction of auxiliary qubits, enabling the effective use of the Ising machine to solve the traffic assignment problem.

In this study, the traffic data is sourced from the open-access T-Drive dataset, which contains GPS trajectories of 10,357 taxis in Beijing recorded between February 2 and February 8, 2008. The dataset includes approximately 15 million GPS points, covering a total distance of 9 million kilometers. To analyze traffic conditions, taxi trajectories during the evening rush hours (16:00–20:59) were mapped onto the road network within Beijing's Third Ring Road. The number of taxis passing through each road segment during this period was counted to determine traffic flow conditions. After aggregating the traffic flow data for all road segments, the initial traffic conditions within the Third Ring Road were established accordingly. For the single origin-destination (OD) pair problem in this study, traffic demand is divided into 160 groups, representing trips from Beijing West Railway Station (origin) to Gulou Street (destination). Each group has three alternative routes, and the impedance function follows the Bureau of Public Roads (BPR) function. The maximum traffic capacity for each road segment is set to 1,600 passenger car units per hour, and a free-flow speed of $16.67\ \mathrm{m\ s^{-1}} (60\ \mathrm{km\ h^{-1}})$ is assumed to calculate the free-flow travel time t_0^s.

Section 9 Traffic Optimization: Selection of Bit Resolution for the *J* Matrix

To demonstrate that a 16-bit resolution for the *J* matrix (Ising coupling matrix) is necessary for solving the traffic optimization problem, we conducted tests using MATLAB SA with 8-bit and 16-bit resolution. For each bit resolution level, the MATLAB SA was executed 100 times, and the average and best energy values were recorded.

For the 8-bit resolution, the average energy value was -1,035,401, and the best energy value was -1,043,732. For the 16-bit resolution, the average energy value was -1,037,821, and the best energy value was -1,046,472. The results indicate that the energy values obtained under 16-bit resolution are closer to those of the IA algorithm, suggesting that higher bit resolution enhances the effectiveness of traffic optimization. Since the OEIM currently supports up to 16-bit resolution, we selected a 16-bit resolution *J* matrix for solving the traffic optimization problem in this experiment.

Section 10 Traffic Optimization: IA Algorithm

The Incremental Allocation (IA) algorithm, derived from Wardrop's first principle, is a non-equilibrium allocation method designed to approximate the user equilibrium (UE) solution[11]. This approach partitions the total traffic demand into *N* sub-demands and sequentially assigns each sub-demand to the route with the lowest total traffic impedance. After each allocation, the impedance of the road network is iteratively updated until all *N* sub-demands have been fully assigned. The accuracy of the IA algorithm is controlled by the parameter *N*; as *N* increases toward infinity, the solution progressively converges to the UE solution.

In this study's single OD pair problem, the IA algorithm is implemented using Dijkstra's algorithm to identify the shortest route, while the total traffic demand is partitioned into 160 sub-demands (corresponding to the 160 groups mentioned earlier). The routes determined by Dijkstra's algorithm are recorded and subsequently used as alternative routes. The pseudocode for the IA algorithm is provided below:

Algorithm 4 *IA*

Input: road network G, including *nodes*, *segments*, etc.
Output: Allocation for N sub-demands *allocation_plan*.
Set total traffic demand, divided into N sub-demands, each with k vehicles.
Use *BPR function* as impedance function.
function update-link (G, *flow*, *BPR*)
for $i = 0$ to $n - 1$ **do**
function find-shortest-path (G, *BPR*, *origin_node*, *destination_node*)
// Use Dijkstra's algorithm to determine the shortest route
function add-flow (G, *shortest_path*, *flow*, k)
// Allocate sub-demands to the shortest path, increasing flow by k
function update-link (G, *flow*, *BPR*)
// Update road network impedance
end for
return *allocation_plan*

The IA method effectively determines specific routes between single OD pairs and provides an optimal solution when the traffic increment granularity is relatively large. In real-world scenarios, traffic flow consists of discrete, non-infinitesimal values, making the IA method's assumptions more consistent with practical conditions. In contrast, Eq. (S4) offers a mathematically optimal solution for the Wardrop model but only describes the final traffic distribution without specifying individual routes between OD pairs. Moreover, its flow values may approach infinitesimal levels, deviating significantly from real-world traffic behavior. For these reasons, this study adopts the IA method to find the reference Ising energy.

Section 11 Traffic Optimization: Comparison of OEIM and SA Algorithms

We used OEIM, MATLAB SA and PySA to solve the real-world traffic optimization problem. It is important to note that both SA algorithms were optimized for their parameters (see Supplementary Section 6). Fig. S3 presents the histogram of Ising energy obtained from 20 runs using OEIM, MATLAB SA, and PySA.

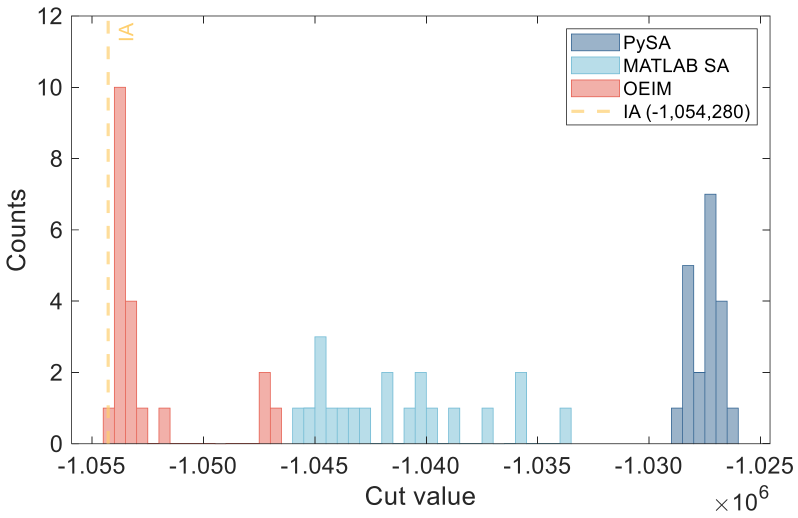

Fig. S3 | The histogram of Ising energy obtained from solving the real-world traffic optimization problem using OEIM, MATLAB SA, and PySA.

Table S2 summarizes the average solving time and average Ising energy values for 20 runs of each method. In terms of computation speed, OEIM outperformed both PySA and MATLAB SA. Regarding solution accuracy, the Ising energy obtained by OEIM was closer to the reference Ising energy, significantly outperforming both MATLAB SA and PySA. Overall, OEIM demonstrated clear advantages over the two SA algorithms in both computation speed and solution accuracy.

	Computation Speed (s)	Ising Energy
OEIM	2.50×10^{-3}	-1,052,495
MATLAB SA	6.49	-1,041,229
PySA	1.08×10^{-1}	-1,027,499

Table S2 | The average solving time and average Ising energy values of OEIM, MATLAB SA, and PySA in solving the real-world traffic optimization problem.

Section 12 Comparison with Other Ising Machine Schemes

Table S2 compares the key performance parameters of the OEIM with other Ising machine schemes. the OEIM possesses a large spin scale, high stability, strong connectivity, high bit-

resolution, and easily achievable operating conditions. Spin scale refers to the number of spins supported in the Ising machine; a larger spin scale means the ability to solve problems with more variables. While the spin scale of OEIM is smaller compared to CIM, it still holds a significant advantage over other approaches. Longer stability time indicates stronger resistance to external disturbance, such as temperature fluctuations and mechanical noise, making it easier to maintain optimal working conditions and achieve high-quality solutions. OEIM demonstrates a clear advantage in stability time compared to CIM and other approaches. Connectivity refers to the extent of arbitrary connections between spins in the Ising machine; the more types of connections that can be realized, the more complex problems the Ising machine can solve. OEIM achieves all-to-all connections between spins, giving it a significant advantage over approaches like D-Wave 2000Q and ROSC, which only allow sparse connections between spins. Since the coupling between OEIM spins is realized through an FPGA, the OEIM can easily change the coupling between spins by loading different Ising coupling matrices J into the FPGA, allowing it to solve various problems without the need for structural changes to the Ising machine. Bit-resolution refers to the system's solving precision in the Ising machine, and OEIM's bit-resolution is significantly higher than that of CIM, spatial-photonic Ising machine, and D-Wave 2000Q, making it better suited to the high precision required by the Ising coupling matrix elements in real-world COPs. Furthermore, OEIM operates stably at room temperature, making it more practical than D-Wave 2000Q, which requires operation in a low temperature environment. Since SPIM requires computer-controlled updates to the SLM state at each iteration, its solving time for COPs is typically on the order of seconds to minutes[12]. In contrast, the OEIM operates on a millisecond timescale, demonstrating a clear advantage in solving speed. Additionally, SBM has seen rapid development in recent years, leveraging multiple cascaded GPUs or FPGAs to solve large-scale Ising problems. However, such approaches require a significant amount of high-performance computing hardware, resulting in high power consumption (typically above 200 W). In comparison, the OEIM achieves large-scale computation using only a single FPGA and a Some low-power optoelectronic devices, highlighting its energy efficiency advantage.

The proposed OEIM leverages the physical advantages of optical waves and microwaves, offering both large-scale and long-term stability. Compared to other Ising machine approaches, it holds greater potential for practical application.

	Representation of spins	Spin Scale	Stability	Connectivity	Bit-resolution	Operation Condition
The proposed OEIM scheme	OEPO	4,096	~5.5 h	All-to-all	16 bits	RT
CIM[7]	DOPO	100,000	~4 s	All-to-all	2 bits	RT
SPIM[12]	Spatial-photonic	16,384	/	All-to-all	8 bits	RT
D-Wave 2000Q[13]	Super-conducting	2,000	~70 μs	Sparse (Chimera)	5-6 bits	<22 mK
ROSC[14]	Transmission gate	1,968	/	Sparse (King's graph)	5 levels	RT
SBM[15]	Digital bits	16,384	/	All-to-all	10 bits	RT

Table S3 | Comparison of Key Performance Parameters between the OEIM and Other Ising Machine Schemes. OEIM: Optoelectronic Ising Machine; CIM: Coherent Ising Machine; SPIM: Spatial-Photonic Ising Machine; ROSC: Ring Oscillator; OEPO: Optoelectronic Parametric Oscillator; DOPO: Degenerate Optical Parametric Oscillator; RT: Room Temperature. Not applicable entries are denoted by "/".

References

1. Cen, Q. Z. et al. Large-scale coherent Ising machine based on optoelectronic parametric oscillator. *Light*: *Science & Applications* **11**, 333 (2022).
2. Miller, R. E., Thatcher, J. W. & Bohlinger, J. D. Complexity of Computer Computations. (New York: Springer, 2013).
3. Goemans, M. X. & Williamson, D. P. Improved approximation algorithms for maximum cut and satisfiability problems using semidefinite programming. Journal of the ACM (JACM) 42, 1115-1145 (1995).
4. Kahruman, S., Kolotoglu, E., Butenko, S. & Hicks, I. V. On greedy construction heuristics for the MAX-CUT problem. International Journal of Computational Science and Engineering 3, 211-218 (2007).
5. McMahon, P. L. et al. A fully programmable 100-spin coherent Ising machine with all-to-all connections. *Science* **354**, 614-617 (2016).
6. Inagaki, T. et al. A coherent Ising machine for 2000-node optimization problems. *Science* **354**, 603-606 (2016).
7. Honjo, T. et al. 100,000-spin coherent Ising machine. *Science Advances* **7**, eabh0952 (2021).
8. Wang, J. N., Wu, C. X. & Zuo, F. More on greedy construction heuristics for the MAX-CUT problem. Print at https://arxiv.org/abs/2312.10895 (2023).
9. NASA. PySA: fast simulated annealing in native python. GitHub. (2025). https://github.com/nasa/PySA.
10. Wardrop, J. G. Road paper. Some theoretical aspects of road traffic research. *Proceedings of the Institution of Civil Engineers* **1**, 325-362 (1952).
11. Saw, K., Katti, B. K. & Joshi, G. Literature review of traffic assignment: static and dynamic. *International Journal of Transportation Engineering* **2**, 339-347 (2015).
12. Wang, R. Z. et al. Efficient computation using spatial-photonic Ising machines with low-rank and circulant matrix constraints. *Communications Physics* **8**, 86 (2025).
13. Hamerly, R. et al. Experimental investigation of performance differences between coherent Ising machines and a quantum annealer. *Science Advances* **5**, eaau0823 (2019).
14. Moy, W. et al. A 1,968-node coupled ring oscillator circuit for combinatorial optimization problem solving. *Nature Electronics* **5**, 310-317 (2022).
15. Tatsumura, K., Yamasaki, M. & Goto, H. Scaling out Ising machines using a multi-chip architecture for simulated bifurcation. *Nature Electronics* **4**, 208-217 (2021).
